# Correlation of Insulin Resistance With Short-Term Outcome in Nondiabetic Patients With ST-Segment Elevation Myocardial Infarction

**DOI:** 10.7759/cureus.33093

**Published:** 2022-12-29

**Authors:** Saja A Al-Ali, Haider A Alidrisi, Abdulameer Hameed

**Affiliations:** 1 Internal Medicine, Basrah Health Directorate, Basra, IRQ; 2 Diabetes and Endocrinology, University of Basrah, College of Medicine, Basra, IRQ; 3 Department of Medicine, University of Basrah, College of Medicine, Basra, IRQ

**Keywords:** • homeostasis model assessment for insulin resistance (homa-ir), mortality, arrhythmias, ejection fraction, tyg index, st-segment elevation myocardial infarction

## Abstract

Background: Obviously, hyperglycemia and insulin resistance (IR) are common in patients with acute ST-segment elevation myocardial infarction (STEMI). Additionally, IR is a substantial risk factor for cardiovascular diseases. The study aims to evaluate the association between IR and short-term outcomes of acute STEMI patients without diabetes mellitus in the form of reperfusion success, the occurrence of heart failure, the development of arrhythmias, and mortality.

Method: A cross-sectional study was done from August 2021 to December 2021 in two cardiology centers in Al-Sadr Teaching hospital and Basrah Oil hospital in Basrah, Southern Iraq. Sixty-one nondiabetic hospitalized patients with acute STEMI were included in the study. Twenty-five (41%) of them received thrombolytics and 36 (59%) were managed with percutaneous transluminal coronary angioplasty. From each patient, a fasting blood sample was taken for calculation of the Homeostasis Model Assessment for IR (HOMA-IR) and triglyceride glucose index (TyG) index. The patients were evaluated within 1-week for (reperfusion success, echocardiography for calculation of the ejection fraction (EF), arrhythmias, and mortality), and within 4-weeks for mortality.

Results: Within the tertile 3 of the HOMA-IR and TyG index, significant higher 4-week mortality (35% and 30%, respectively). Pearson correlation also showed significant and negative correlations between both HOMA-IR and TyG index values and EF. While reperfusion success, arrhythmias, and 1-week mortality did not correlate significantly with both HOMA-IR and TyG index.

Conclusion: IR as defined by HOMA-IR and TyG index was significantly associated with poor outcomes in patients with acute STEMI in the form of EF<55 and 4-week mortality.

## Introduction

Acute ST-segment elevation myocardial infarction (STEMI) is a condition in which transmural myocardial ischemia causes myocardial injury or necrosis [1]. In hospitalized patients with STEMI, hyperglycemia is prevalent and is linked to poor clinical outcomes. Multiple factors, such as underlying medical problems, pathophysiological stresses, and medications, contribute to hyperglycemia in hospitalized STEMI patients. In both diabetic and nondiabetic patients, the development of temporary insulin resistance (IR) is a known cause of hyperglycemia [2].

IR is described as “a state of a cell, tissue, or organism in which more insulin is required to elicit a quantitatively normal response” [3]. Undoubtedly, in patients with STEMI treated with primary percutaneous coronary intervention (PCI), acute IR is a dynamic phenomenon linked to the development of myocardial and microvascular dysfunction, as well as a greater final infarct size [4]. In addition, IR is a component of metabolic syndrome, and it is a significant risk factor for the development of cardiac and vascular problems [5,6]. Some research studies suggest that IR has direct proatherogenic effects as well as direct harmful consequences on cardiac tissue [7,8]. The Homeostatic Model Assessment (HOMA) index is a simple and inexpensive IR marker. It was recently proven to be feasible for evaluating IR in STEMI patients when compared to euglycemic hyperinsulinemic clamp [9].

The triglyceride glucose (TyG) index, which is derived from fasting triglyceride (TG) and fasting plasma glucose (FPG) levels, has been proposed as a reliable surrogate marker of IR. Numerous studies have found a positive correlation between the TyG index and cardiovascular risk, including systematic arterial stiffness, carotid atherosclerosis, coronary artery calcification, coronary artery stenosis, symptomatic coronary artery disease, hypertension, and metabolic syndrome [10,11]. Furthermore, growing evidence has indicated that the TyG index is related to morbidity and mortality of cardiovascular disease in the general population and many patient cohorts, including patients with and without diabetes [12,13].

The study aims to evaluate the association between IR and short-term outcomes in patients with acute STEMI without diabetes mellitus in the form of reperfusion success, the occurrence of heart failure, development of arrhythmias, and mortality.

This article was previously presented as a meeting abstract at the annual scientific meeting of the Endocrine Society “ENDO 2022” on June 11-14, 2022.

## Materials and methods

Study population

A cross-sectional study was done from the 1st of August/2021 to the 1st of December/2021 in two cardiology centers in Al-Sadr Teaching hospital and Basrah Oil hospital in Basrah, Southern Iraq. Clinical data were collected through direct interview with the patients and revising the medical records for the laboratory data and electrocardiographic (ECG) findings.

Inclusion criteria:

Adult patients admitted after a diagnosis of acute STEMI as defined by characteristic symptoms of myocardial ischemia in association with ECG findings of persistent new ST-segment elevation at the J-point in two contiguous leads with the cut-points: ≥0.1 mV in all leads other than leads V2-V3. For leads V2-V3: ≥2 mm in men ≥40 years, ≥2.5 mm in men <40 years, or ≥1.5 mm in women regardless of age, and subsequent release of biomarkers of myocardial necrosis [14].

Exclusion criteria

The following patients were excluded from the study: Type 1 or 2 diabetes mellitus, cardiogenic shock, and/or cardiac arrest on presentation, any previous ischemic cardiovascular event including MI, unstable angina, and stroke, left ventricular systolic dysfunction, diuretic treatments, chronic kidney disease, chronic liver disease, dyslipidemia currently on either statin, and/or fibrate treatments.

Sixty-one patients were included in the study, 48 men and 13 women. Informed consent was obtained from the patients. Additionally, the study was approved by the training and the Human Development Center in Basrah Health Directorate, and ethical consideration was waived from the ethical committee in Basrah Medical College. The investigation followed the principles outlined in the Declaration of Helsinki. The ethical committee of the University of Basrah, College of Medicine approved the study protocol (2020-03040856).

Interventions

All the patients were evaluated and received treatments in the centers as recommended by guidelines [14]. Reperfusions were done on the patients according to their availability. Twenty-five (41%) patients received thrombolytics and 36 (59%) were managed with percutaneous transluminal coronary angioplasty (PTCA).

Measurement of IR

From each patient, a fasting 10 mL blood sample was taken, put in a clot activator tube, centrifuged and the serum separated immediately and analyzed within 1-7 hours. The samples were not specifically collected for the study, and were taken as part of routine evaluation and management of the included patients. The fully automated chemiluminescence immunoassay kit Cobas e411 analyzer series/Roche Diagnostics, Germany, was used for the assessment of insulin level (reference value from 2.6 to 24.9 μU/mL (18.05 to 172.9 pmol/L)). Glucose and TG were measured by INTEGRA 400 PLUS (COBAS).

The degree of IR was assessed using two methods: HOMA-IR = (fasting plasma glucose (mg/dL) × fasting plasma insulin (μU/mL)/405). [9] And the TG glucose index TyG index = (ln [Fasting TG (mg/dL) x Fasting plasma glucose (mg/dL)]/2). [15].

 One-week outcome

Within the first week and during hospitalization, the evaluations included.

Successful recanalization: PTCA success was defined as final thrombolysis in myocardial infarction (fTIMI) flow 3, while failure was defined as an fTIMI 0 to 2 flow. Thrombolytics success was assessed by the following parameters [14]:

· Clinically as relief from chest pain, ECG-ST-segment regression > 50%, cardiac enzymes (early flushing of intra myocytic creatine phosphokinase into the systemic circulation and hence early peaking of creatine phosphokinase MB (<1 hour instead of 24 h)), reperfusion arrhythmias (Accelerated Idioventricular Rhythm-Less specific), infract-related artery (IRA) patency by coronary angiogram, and distal TIMI flow/myocardial blush score/TIMI frame count.

· Any tachyarrhythmias (supraventricular and ventricular) and bradyarrhythmia (any degree of heart block) other than premature atrial and/or ventricular beats.

· First-week mortality and other mechanical cardiac complications in the form of cardiogenic shock, rupture of the left ventricular free wall, rupture of the interventricular septum, and acute mitral regurgitation.

· Before discharge, two-dimensional echocardiography was done on every patient for calculation of the ejection fraction (EF) (Philips iE33).

Four-week outcome

After the end of the four-week post-STEMI, each patient received a phone call for further assessment of cardiovascular-related events (acute coronary syndrome, heart failure, and stroke) and mortality.

Statistical analysis

Data were analyzed using the Statistical Package for Sciences (SPSS), version 26.0 (IBM SPSS Statistics for Windows, Armonk, NY). Quantitative data were presented as mean ± standard deviation, and qualitative data were presented as numbers and percentages (N (%)). The HOMA-IR was categorized into three tertiles (tertile 1 < 2.5, tertile 2 2.5 - <5, and tertile 3 ≥5). TyG index was also categorized into three tertiles (tertile 1 <4.73, tertile 2 4.73 - <4.87, and tertile 3 ≥4.87). The Fisher Exact test was used to test the associations between both HOMA-IR and TyG index tertiles, and the STEMI outcomes. Pearson’s correlation was used to assess the strength and the significance of the correlations between both HOMA-IR and TyG index, and post-STEMI EF. The Chi-square test and Fisher Exact test were used to test the association between the patients’ characteristics in the form of (age, gender, smoking, ECG findings, and reperfusion type), and post-STEMI EF, 4-week mortality. For all of the above comparisons, a P value of less than 0.05 was considered statistically significant.

## Results

Table 1 summarizes the general characteristics of the study patients with STEMI.

**Table 1:** General characteristics of the study patients (N = 61). TG: triglyceride; FPG: fasting plasma glucose; HOMA: homeostatic model assessment for insulin resistance; TyG index: triglyceride and glucose index; PTCA: percutaneous transluminal coronary angioplasty

Variable		Mean ± standard deviation or N (%)
Age (years)		54.6 ± 11.6
Age ≥ 55 years		30 (49.2)
Gender (men)		48 (78.7)
Current smoking		47 (77)
Hypertension		19 (31.1)
TG (mmol/l)		1.80 ± 0.84
FPG (mmol/l)		5.84 ± 0.58
Insulin (µU/mL)		14.8 ± 8.2
HOMA-IR		3.8 ± 2.2
Tertile 1 <2.5		18 (29.5)
Tertile 2 2.5 - <5		23 (37.7)
Tertile 3 ≥5		20 (32.8)
TyG index		4.8 ± 0.2
Tertile 1 <4.73		21 (34.4)
Tertile 2 4.73- <4.87		20 (32.8)
Tertile 3 ≥4.87		20 (32.8)
	Anterior and anterolateral	20 (32.8)
	Anterior and inferior	4 (6.6)
Reperfusion	Thrombolytics	25 (41)
	Primary PTCA	36 (59)

Within the first week of the follow-up of the patients, the mean EF was 58.5 ± 9.8% with nine (14.8%) having EF of less than 55%. Nine patients (14.8%) had non-successful recanalization, and three developed arrhythmias (two atrial fibrillation (AF), and one ventricular fibrillation (VF)). None of the patients died during the first week, and eight (13.1%) died over a 4-week follow-up, as shown in Table 2.

**Table 2:** Short-term outcome of the study patients with STEMI (N = 61). EF: ejection fraction; AF: atrial fibrillation; VF: ventricular fibrillation

	Mean ± standard deviation or N (%)
EF %	58.5 ± 9.8
EF < 55%	9 (14.8)
Non-successful recanalization	9 (14.8)
Arrhythmias	3 (4.9)
AF	2 (3.3)
VF	1 (1.6)
1-week mortality	0
4-week mortality	8 (13.1)

**Figure 1:**
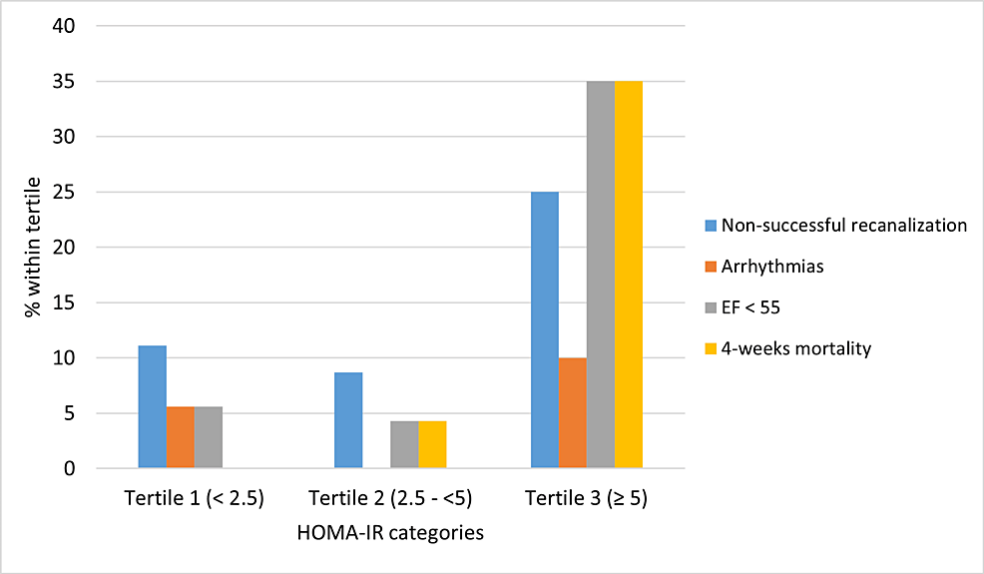
Association between HOMA-IR severity categories and outcomes of acute STEMI. Fisher’s Exact Test value and P value for the study outcomes including non-successful recanalization (2.28, P = 0.3), arrhythmias (2.24, P = 0.2), EF <55 (8.1, P = 0.01), and 4-week mortality (10.5, P = 0.002). HOMA-IR: homeostatic model assessment for insulin resistance; STEMI: ST-segment elevation myocardial infarction; EF: ejection fraction

As shown in Figure 1, Within the tertile 3 of HOMA-IR, the patients were significantly more likely to have EF < 55% (35%), and significantly higher 4-week mortality (35%). Also, there was a numeric but non-significant higher non-successful revascularization rate.

**Figure 2:**
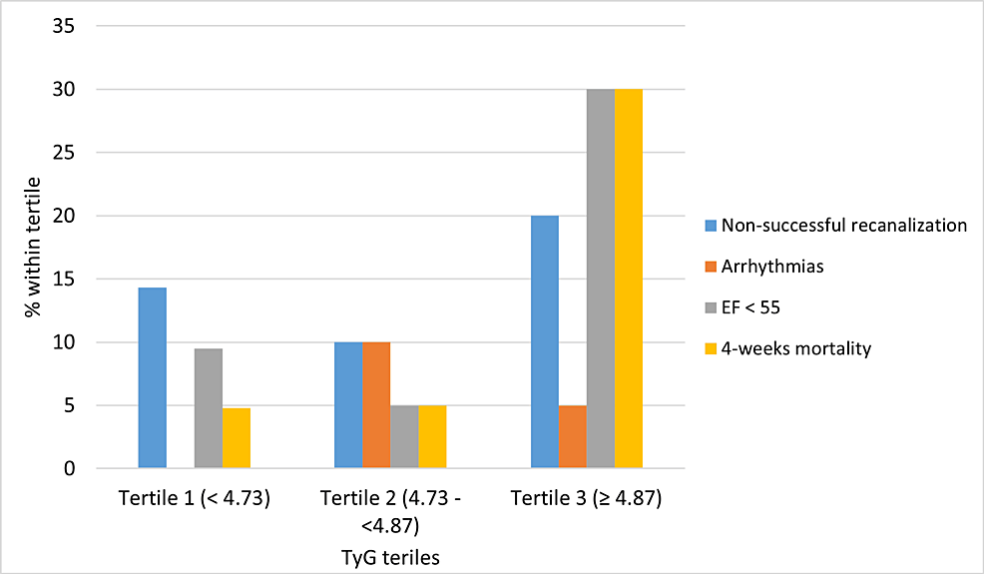
Association between TyG index severity categories and outcomes of acute STEMI. Fisher’s Exact Test value and P value for the study outcomes including non-successful recanalization (0.8, P = 0.7), arrhythmias (2.02, P = 0.3), EF <55 (4.8, P = 0.09), an and 4-weeks mortality (6.1, P = 0.03). TyG index: triglyceride glucose index; STEMI: ST-segment elevation myocardial infarction; EF: ejection fraction

In Figure 2, within the TyG index in the tertile 3 (≥ 4.87), there was a significant higher 4-week mortality (30%). Also, there was a numeric but non-significant higher likelihood to have EF <55% and a non-successful revascularization rate.

**Figure 3:**
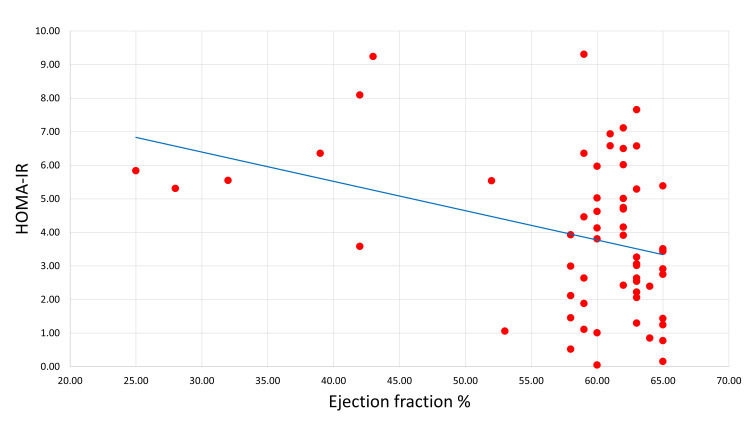
Pearson correlation between early post-STEMI ejection fraction value and HOMA-IR. (R = -0.3, P = 0.006). HOMA-IR: homeostatic model assessment for insulin resistance; STEMI: ST-segment elevation myocardial infarction

Figure 3 shows the Pearson correlation between the early post-STEMI EF and the degree of IR by HOMA-IR. There was a significant and negative correlation between HOMA-IR and EF.

**Figure 4:**
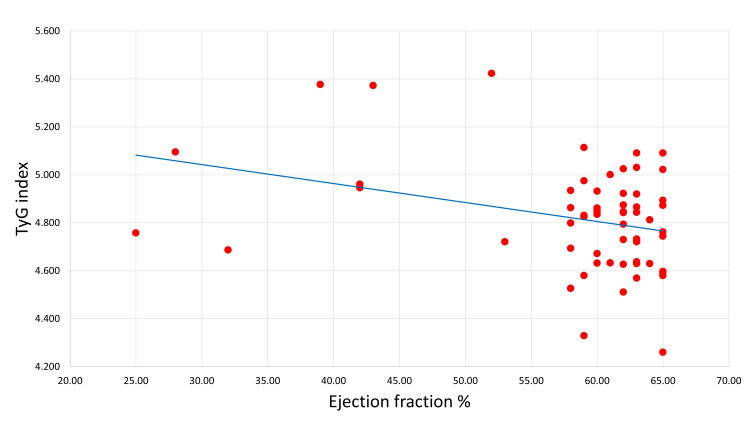
Pearson correlation between early post-STEMI ejection fraction value and TyG index. (R = -0.32, P = 0.01). TyG index: triglyceride glucose index; STEMI: ST-segment elevation myocardial infarction

Figure 4 shows the Pearson correlation between the early post-STEMI EF and the degree of IR by the TyG index. There was a significant and negative correlation between the TyG index and EF.

From the other cofounders in the study, EF <55% and 4-week mortality were significantly higher in women vs men and non-smokers vs smokers, as shown in Table 3. While age, ECG findings, and thrombolytics vs PTCA did not correlate significantly.

**Table 3:** Correlation between different patients’ cofounders, post-STEMI EF, and 4-week mortality. *P value by Fisher’s Exact test, all other correlations by Chi-Square test. STEMI: ST-segment elevation myocardial infarction; ECG: electrocardiography; PTCA: percutaneous transluminal coronary angioplasty

	EF N (%)		4-week mortality N (%)	
	< 55%	≥ 55%	Died	Alive
Age < 55 years	3 (9.7)	28 (90.3)	3 (9.7)	28 (90.3)
Age ≥ 55 years	6 (20)	24 (80)	5 (16.7)	25 (83.3)
P value	0.2		0.4	
Men	4 (8.3)	44 (91.7)	3 (6.3)	45 (93.8)
Women	5 (38.5)	8 (61.5)	5 (38.5)	8 (61.5)
P value	0.007		0.002	
Smoker	4 (8.5)	43 (91.5)	4 (8.5)	43 (91.5)
Non-smoker	5 (35.7)	9 (64.3)	4 (28.6)	10 (71.4)
P value	0.01		0.05	
ECG findings				
Inferior	5 (13.5)	32 (86.5)	5 (13.5)	32 (86.5)
Anterior and anterolateral	4 (20)	16 (80)	3 (15)	17 (85)
Anterior and inferior	0	4 (100)	0	4 (100)
P value*	0.8		1.0	
Thrombolytics	4 (16)	21 (84)	4 (16)	21 (84)
PTCA	5 (13.9)	31 (86.1)	4 (11.1)	32 (88.9)
P value	0.8		0.5	

## Discussion

Early post-STEMI complications are significantly associated with increased values of HOMA-IR and TyG index. Patients with EF < 55%, post-STEMI arrhythmia, and unsuccessful recanalization were mainly seen in the tertile 3 of the scoring system which represents the more severe form of IR. Insulin lowers platelet aggregation and fibrinogen generation, and has anti-inflammation and antioxidant activities. These have a favorable effect on endothelial action and vascular wall function according to in vitro and in vivo evidence [16,17]. All of these benefits would be attenuated in IR situations, which could explain why IR STEMI patients have a worse prognosis. Furthermore, as implied by the relationship between the HOMA index, TyG index, and the early post-STEMI complications, IR might be seen as a contributor to the complex metabolic derangements that characterize the initial stages of STEMI patients [18]. Gruzdeva et al. did successive HOMA-IR index assessments for the patients that had done primary PCI and they found that whether the IR was transient and related to stress hormone or remain for a period after the PCI, it is usually associated with increased leptin level and correlated with restenosis [19].

Our study found that the major complication which is associated with tertile 3 HOMA-IR and TyG index among survived patients is the reduction in EF to less than 55%. For decades, there has been a strong relationship between heart failure and systemic IR in humans. Myocardial insufficiency and heart failure are commonly preceded by systemic IR, implying that a changed metabolic state causes myocardial malfunction and heart failure [20]. Botker et al. became the first to demonstrate cardiac IR in metabolic syndrome patients in 1997 [21]. Cardiac IR can develop as a result of systemic IR [22].

This study linked unsuccessful thrombolytic therapy with a more severe form of IR, similar findings were obtained by Calleja et al., in 2011 when they found that IR was associated with poor response to thrombolysis in ischemic stroke treatment [23]. In this setting, IR can counteract the effects of systemic thrombolysis through a variety of methods. First, people with severe IR have higher levels of fibrinolysis blockers in their blood, such as plasminogen activator inhibitor 1, which could indicate a problem with natural fibrinolysis [24]. Second, IR may change the composition of the offending clot, making it denser and refractory to lysis [25]. Furthermore, clots collected from patients with metabolic syndrome are made up of denser fibers and have longer lysis durations than those obtained from people who do not have an elevated IR [26]. Despite that, there is no significant difference in the percentage of patients who had heart failure (which is the major complication that is associated with IR) and the proposed method of recanalization whether PCI or thrombolytic therapy.

In this study, the reported AF cases have an increased TyG index above 4.73. A similar finding explored by Ling et al., on 549 STEMI patients in Wuhu, China who were concluded that during admission, the TyG index is an independent predictor of AF. Furthermore, those who had AF while in the hospital had a much worse outcome following discharge [27]. Hyperinsulinemia is thought to play a role in the stimulation of the sympathetic system as well as the renin-angiotensin-aldosterone pathway, resulting in atrial neuronal remodeling and increased vulnerability to AF [28]. According to Maria et al., IR suppresses atrial glucose transporter production, which contributes to the formation of metabolic circumstances favorable to the development of AF [29].

The study has limitations. First, IR measurement whether by HOMA index or TyG index was measured once without monitoring the IR status after 4 weeks. Second, the effect of various treatments given to the patient during the hospitalization and after discharge were not analyzed on the clinical outcomes. Finally, the small number of complications made the interpretation of the results and their subsequent correlation to confounders difficult.

## Conclusions

IR as defined by HOMA-IR and TyG index was significantly associated with poor outcomes in acute STEMI patients without diabetes mellitus in the form of EF<55% and 4-weeks mortality. Both HOMA-IR and TyG indexes have significant negative correlations with the early post-STEMI EF. Other outcomes, including reperfusion success, arrhythmias, and 1-week mortality did not correlate significantly with IR. While other patients’ characteristics in the form of age, gender, smoking status, myocardial infarcted wall, and the method of recanalization did not correlate significantly.

While it is simple and easy to measure FPG, insulin, and TG, we recommend further studies assessing IR in patients with acute STEMI as a marker for poor outcomes in those critically ill patients.
